# A Diet Enriched in Docosahexanoic Acid Exacerbates Brain Parenchymal Extravasation of Apo B Lipoproteins Induced by Chronic Ingestion of Saturated Fats

**DOI:** 10.1155/2012/647689

**Published:** 2011-11-10

**Authors:** Menuka M. Pallebage-Gamarallage, Virginie Lam, Ryusuke Takechi, Susan Galloway, John C. L. Mamo

**Affiliations:** ^1^Curtin Health Innovation Research Institute, Curtin University of Technology, Perth, Western Australia 6845, Australia; ^2^Centre for Metabolic Fitness, Australian Technology Network Universities, Perth, Western Australia 6845, Australia

## Abstract

Chronic ingestion of saturated fatty acids (SFAs) was previously shown to compromise blood-brain barrier integrity, leading to brain parenchymal extravasation of apolipoprotein B (apo B) lipoproteins enriched in amyloid beta. In contrast, diets enriched in mono- or polyunsaturated (PUFA) oils had no detrimental effect. Rather, n3 and n6 oils generally confer protection via suppression of inflammation. This study investigated in wild-type mice if a PUFA diet enriched in docosahexanoic acid (DHA) restored blood-brain barrier integrity and attenuated parenchymal apo B abundance induced by chronic ingestion of SFA. Cerebrovascular leakage of apo B was quantitated utilising immunofluorescent staining. The plasma concentration of brain-derived S100*β* was measured as a marker of cerebrovascular inflammation. In mice fed SFA for 3 months, provision thereafter of a DHA-enriched diet exacerbated parenchymal apo B retention, concomitant with a significant increase in plasma cholesterol. In contrast, provision of a low-fat diet following chronic SFA feeding had no effect on SFA-induced parenchymal apo B. The findings suggest that in a heightened state of cerebrovascular inflammation, the provision of unsaturated fatty acids may be detrimental, possibly as a consequence of a greater susceptibility for oxidation.

## 1. Introduction

Accumulating evidence supports the hypothesis that dietary behaviour and in particular ingestion of fats contribute to Alzheimer's disease (AD) onset and progression. The work in [1] reported that the prevalence of sporadic and late-onset AD in >65 years of age subjects correlated with fat intake and was higher in Western countries, compared to Africa or Asia. Population and clinical studies also suggest that regular consumption of saturated fatty acids (SFAs) and trans-fatty acids as well as cholesterol is synergistically and positively associated with increased risk of AD [1, 2] through mechanisms that include dyslipidemia, endothelial dysfunction, inflammation, and oxidative stress. In contrast, populations with greater consumption of fats as poly- or mono-unsaturated oils (PUFA and MUFA, resp.) have lower prevalence of AD and vascular based dementias [1–4], probably as a consequence of lower levels of systemic inflammation [5–7]. 

In AD, chronic inflammation leading to neuronal loss appears to be primarily associated with cerebrovascular and brain parenchymal deposits of amyloid beta (A*β*) [8]. Derived from the amyloid precursor protein, A*β* is the predominant component of  “amyloid” (or senile) plaques [9, 10]. Key triggers of cerebrovascular amyloidosis are thought to include enhanced proteolytic processing of the precursor protein on the plasma membrane of neuronal cells [11–13], a phenomenon more common in early-onset AD. In addition, fibrillar formation of A*β* and deposition upon extracellular matrices may also reflect decreased degradation and efflux by epithelial cells of the choroid plexus [14, 15]. Alternatively, cerebral parenchymal A*β* load may be exacerbated if cerebrovascular integrity is compromised and blood-to-brain delivery of peripheral A*β* is increased [16, 17]. Moreover, the latter typically results in the activation of astro-glial cells and oxidation of proteins and lipids [18, 19]. 

Significant plasma A*β* is found associated with triglyceride-rich lipoproteins (TRLs) and cell culture and immunohistochemical studies confirm secretion of A*β* as a TRL from hepatocytes and absorptive epithelial cells of the small intestine [20–22]. In humans there is a transient increase in plasma A*β* concentration, following the consumption of a mixed lipid meal and kinetic studies in vivo showing that A*β* serves as a regulating apolipoprotein of TRLs [23]. However, several lines of evidence suggest that persistent disturbances in the TRL-A*β* pathway may contribute to AD risk. In three strains of amyloid transgenic mice, secretion into plasma of TRL-A*β* was strongly associated with onset and progression of amyloidosis [24]. Moreover, significant cerebrovascular disturbances were reported preceding plaque formation in amyloid transgenic mice [25]. Consistent with the concept of disease induction in response to exaggerated exposure, subjects with AD were reported to have significantly elevated plasma TRL-A*β* concomitant with evidence postprandial dyslipidaemia [26]. Moreover, in human cadaver and in transgenic-amyloid mice brain specimens, significant apolipoprotein B (apo B) immunoreactivity colocalized with early diffused amyloid plaque [27, 28].

To explore directly the hypothesis of a dietary fat modulation of TRL-A*β* and cerebrovascular integrity axis, wild-type (WT) mice were fed diets enriched in either SFA, MUFA, or PUFAs [28]. Within 12 weeks of dietary intervention, mice maintained on the SFA diet showed substantial parenchymal extravasation of plasma proteins including apo B lipoproteins enriched with A*β*. The endothelial tight junction protein occludin was substantially attenuated in SFA-fed mice concomitant with substantially increased enterocytic abundance of A*β* [28, 29]. In contrast, mice maintained on either the MUFA or PUFA diets had no cerebrovascular aberrations and penetration of plasma proteins in these two groups was comparable to low-fat- (LF-) fed controls. 

The potential cytotoxic properties of SFA are established and the principal mechanisms include mitochondrial respiratory burst resulting in oxidative stress and endoplasmic reticulum dysfunction [30]. Polyunsaturates on the other hand and in particular docosahexanoic acid (DHA) generally antagonise the effects of SFA and are purported to confer cytoprotection because of potent anti-inflammatory effects [5, 31–33]. However, unsaturated fatty acids such as DHA are highly susceptible to lipid peroxidation and if inflammation is already established, then oxidative damage may be paradoxically amplified with the provision of unsaturated fatty acids such as DHA. To explore the hypothesis that polyunsaturated fatty acids confer benefit and not risk in a cerebrovascular proinflammatory state, we explored parenchymal extravasation of apo B lipoproteins in WT mice initially maintained on an SFA-enriched diet for 3 months, followed by randomization to either an LF- or a PUFA-enriched diet for 2 months. 

## 2. Methods

### 2.1. Animals and Diet Conditions

The animal housing, handling, and experimental procedures described for this study were approved by the Curtin University Animal Experimentation and Ethics Committee. Six-week-old female WT mice (C57BL/6J) were housed in groups and randomized into their respective diets (*n* = 6 mice per group). All mice were maintained on a 12 h light and dark cycle room, at 22°C and with free access to water and food. 

Mice were fed an SFA diet (SF07-050, Glen Forrest Stockfeeders, Glen Forrest, Western Australia) for 3 months and then randomised to receive either an LF (AIN-93M, Glen Forrest Stockfeeders), PUFA-enriched (SF07-049, Glen Forrest Stockfeeders), or maintained on SFA for a further 2 months. Fatty acid composition for each diet is shown in Table 1. Digestible energy for the SFA diet was 18.8 MJ/kg and contained palmitic (16 : 0) and stearic (18 : 0) as the principle saturated fats (13%, w/w). The LF diet contained 4% (w/w) as total fat and 15.1 MJ/kg of digestible energy and only <1% of total digestible energy from lipids. The PUFA diet contained 8.22% (w/w) of DHA and 2.00% (w/w) eicosapentaenoic acid (EPA) sourced from NUMEGA fish oil. The diet generated 18.8 MJ/kg of digestible energy where 40% of energy derived from lipids. All diets had sufficient essential fatty acids.

### 2.2. Tissue Collection and Sample Preparation

Blood and brain samples were collected as previously described by Takechi et al. [28]. Mice were anaesthetised with pentobarbitone (45 mg/kg i.p.), and exsanguinated by cardiac puncture. Blood was collected into K-2 EDTA tubes and stored in ice. Plasma was separated by short speed centrifugation at 4°C and stored at −80°C. 

Brain tissues were carefully isolated, washed with chilled phosphate buffer saline (PBS, pH 7.4), right hemispheres were separated, and fixed in 4% paraformaldehyde for 24 h. The tissues were then cryoprotected with 20% sucrose solution at 4°C for 72 h, frozen in isopentane with dry ice and stored at −80°C. For histology and fluorescence microscopy, serial cryosections of 18 *μ*m were cut from the right cerebral hemispheres for each mouse and mounted on Polysine slides (LabServ, Australia). 

### 2.3. Cerebral Apo B Immunofluorescence

Cerebrovascular leakage of apo B was detected as previously described [28]. Brain cryosections (18 *μ*m) were air-dried for 30 min, rehydrated with PBS and incubated in blocking serum (10% goat serum) for 30 min prior to the application of the antibodies. Cerebral apo B was detected by overnight incubation with the primary antibody polyclonal rabbit anti-apo B (ab20737, Abcam, Cambridge, UK) at 1 : 500 dilution, at 4°C. Postovernight incubation and washing with PBS, primary antibody was labelled at room temperature with the secondary goat anti-rabbit IgG-Alexa 488 fluorochrome conjugate (A-11034, Invitrogen) for 2 h. The sections were then washed with PBS and the nuclei were counterstained with DAPI (1 : 1000) for 5 min at room temperature. Thereafter, the stained sections were mounted with antifade mounting medium. Primary antibody was replaced with buffer or an irrelevant serum for negative control tissues.

### 2.4. Quantitative Immunofluorescent Imaging and Analysis

Digital images for photomicroscopy were acquired through AxioCam HRm camera (Carl Zeiss, Germany) with an AxioVert 200 M inverted microscope by Zeiss (Germany) at ×200 magnification (Plan Neofluar ×20 objective, 1.3 numerical aperture). Three-dimensional (3D) images were captured through ApoTome optical sectioning methodology (Carl Zeiss). Each 3D image consisted of 6–10 two-dimensional (2D) images and the distance between Z-stack slices was 1.225 *μ*m optimised by Nyquist. A minimum of nine 3D images were randomly captured per mouse, which include 5 images within the cortex (CTX) and 2 images each from brainstem (BS) and hippocampal formation (HPF). 

Cerebrovascular leakage of plasma protein apo B was quantified within the CTX excluding the hippocampus, BS, and HPF. The pixel intensity of the protein of interest for each 3D image was calculated utilising the automated optical intensity measurement tool in Volocity (Software version 5.5, Perkin Elmer, Melbourne, Australia) and expressed as per unit volume. The investigator was blinded during imaging and quantitation.

### 2.5. Plasma Cholesterol, Triglyceride, and NEFA

Plasma cholesterol and triglycerides were determined in duplicate by enzymatic assays (Randox Laboratories LTD, UK). Nonesterified fatty acids (NEFAs) were determined with NEFA-C (ASC-ACOD method, Wako Pure Chemical Industries, Osaka, Japan). 

### 2.6. Plasma S100*β* Analysis

Plasma S100*β* is used as a marker of cerebrovascular inflammation and was measured using ELISA kits according to manufacturers' instructions (CosmoBio, Tokyo, Japan). Plasma S100*β* was measured with 30 *μ*L of plasma sample or standard and incubated in precoated microtitre well plates at 4°C overnight. Plates were then incubated with biotinylated secondary antibody and Streptavidin-HRP for 2 h, each. Colour generated with substrate and optical density determined at 492 nm. After adjusting for sample dilution, final concentrations of plasma S100*β* were extrapolated from standard curve.

### 2.7. Statistical Analysis

This study utilised *n* = 6 mice per group and minimum of nine 3D images were captured per mouse for detection of apo B leakage. Each 3D image was constructed by stacking of sequential 2D images, therefore generating 324–540 two-dimensional images per group. All data was analysed by either parametric or nonparametric one-way Analysis of Variance to assess the main effects of LF and DHA on dietary SFA-induced blood-brain barrier (BBB) dysfunction and their two-way interactions. Post-hoc comparison of means was done if the associated main effect or interaction was statistically significant within the Analysis of Variance procedure. *P* values < 0.05 were considered statistically significant.

## 3. Results

**Figure 1:**
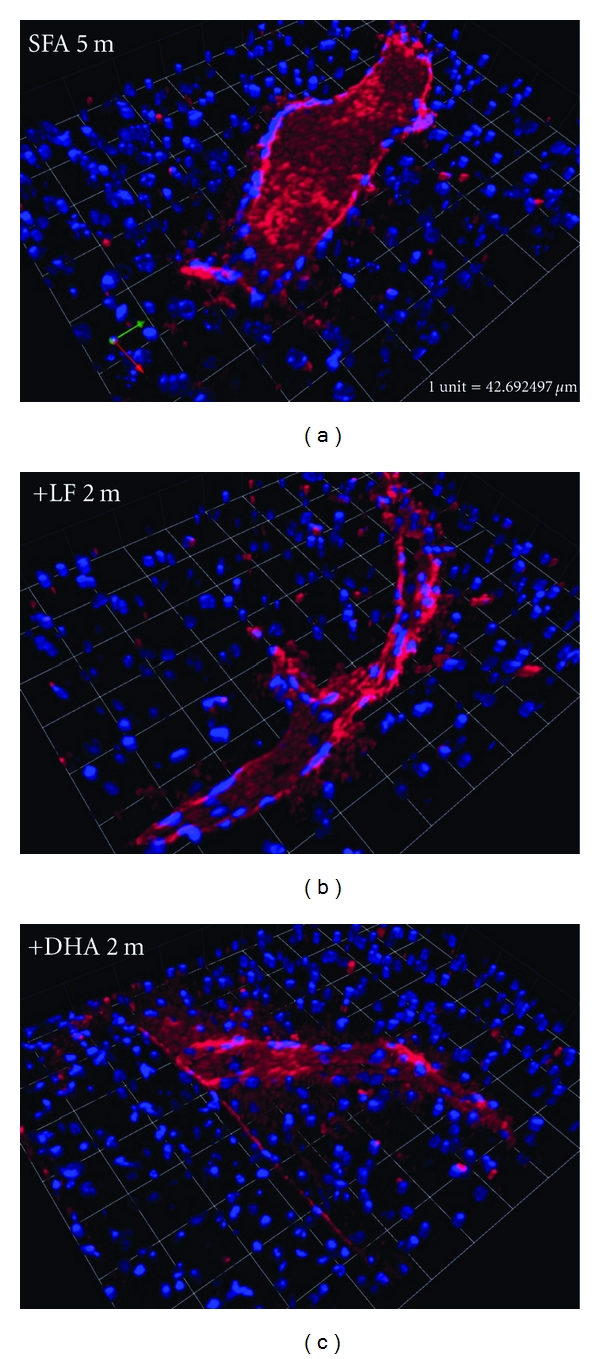
Three-D immunofluorescent staining of cerebral extravasation of apo B. Parenchymal leakage of apo B lipoproteins (red) is observed surrounding the cerebral microvessels. Nuclei are shown in blue. The 3D images are from mice maintained on saturated-fat diet for 5 months (SFA 5 m) and SFA fed mice randomised to LF (+LF 2 m) and DHA (+DHA 2 m) diet for further 2 months. Scale: 1 unit = 42.7 *μ*m.

**Figure 2:**
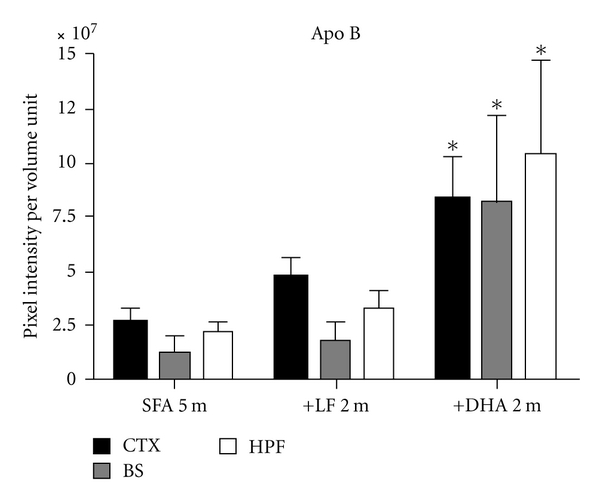
Three-dimensional (3D) quantitative analysis of apo B leakage in C57BL/6J mouse brain. Pixel intensity of apo B lipoproteins surrounding the cerebrovasculature was quantitated in 3D images from mice maintained on saturated-fat diet for 5 months (SFA 5 m) and SFA-fed mice randomised to LF (+LF 2 m) and DHA (+DHA 2 m) diet for further 2 months. The extent of apo B pixel intensity was measured in the cortex (CTX), brain stem (BS), and hippocampal formation (HPF) and expressed as per unit volume. The bars represent mean intensity and standard error of mean, where *P* < 0.05 considered statistically significant (* Kruskal Wallis *t*-test). The C57BL/6J mice randomised to DHA diet (+DHA) had elevated apo B intensities in all regions of the brain.

**Figure 3:**
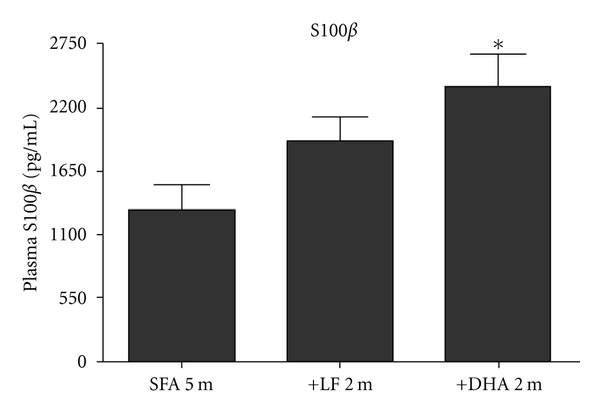
Effect of dietary fatty acids on plasma S100*β* concentration in C57BL/6J mice. The brain abundant protein S100*β* was measured in plasma as a surrogate marker of BBB leakage. High plasma S100*β* concentration in SFA-fed mice correlate with significant BBB dysfunction. In comparison to SFA 5 m group, plasma S100*β* was significantly increased in mice switched to the DHA diet (+DHA 2 m). The bars represent mean plasma concentration (pg/mL) and standard error of mean, where *P* < 0.05 considered statistically significant (* one-way ANOVA).

We confirm previous studies showing significant CTX > BS > HPF extravasation of apo B lipoproteins in WT mice maintained on an SFA diet for a total of 5 months (3 months plus randomization to SFA for an additional 2 months: Figures 1 and 2). Mice randomized to an LF diet following 3 months of SFA feeding had comparable levels of parenchymal apo B lipoprotein abundance to mice maintained on SFA feeding alone (Figures 1 and 2). However, in mice randomized to the DHA-enriched diet following 3 months of SFA feeding, parenchymal apo B abundance was markedly increased (Figures 1 and 2). The cerebrovascular effects of the DHA diet occurred commensurate with a 2-fold increase in plasma cholesterol compared to mice maintained on the SFA diet (Table 2). In contrast, the LF diet had no significant impact on plasma lipid homeostasis. Disturbances in BBB integrity and function were supported by the findings of substantially increased plasma S100*β* in the SFA→DHA mice compared to SFA alone (Figure 3). The protein S100*β* is commonly used as a surrogate marker of brain-to-blood leakage. The S100*β* is a cytokine produced exclusively by the astrocytes of the central nervous system. Following randomization, differences in food consumption were identified. Mice maintained on SFA or randomized to the LF consumed on average 3 g/day, whereas consumption of the DHA-enriched diet was reduced to 2 g/day. The lower caloric intake of mice on the DHA enriched diet resulted in a slower rate of growth following randomization (Table 2).

## 4. Discussion 

This study was designed to explore if provision of a diet enriched in DHA attenuated cerebrovascular dysfunction induced by chronic ingestion of an SFA diet. The primary outcome measure was to determine the abundance of brain parenchymal apo B lipoproteins that transport significant endogenous A*β*. Cerebral capillary vessels normally have tightly apposed endothelial cells that ordinarily prevent transport of plasma proteins and macromolecules [34]. 

The primary finding of this study showed that provision of a PUFA diet principally enriched in DHA exacerbated brain parenchymal extravasation of apo B lipoproteins that had been initially induced by chronic ingestion of SFA. Previous studies exploring the effect on cerebrovascular integrity and function by the SFA and PUFA diets described here, as well as an MUFA-enriched diet, showed in C57BL/6J mice that only the SFA diet induced parenchymal accumulation of apo B lipoproteins [28]. Therefore, the paradoxical effects of the PUFA diet are likely to be a consequence of amplification of proinflammatory pathways induced as a consequence of chronic SFA ingestion. Consistent with this concept, SFA-fed mice randomized to an LF diet showed similar parenchymal apo B abundance and plasma S100*β* as mice that were maintained on SFA alone. 

Several studies have provided evidence of a vasoactive role of A*β*, with pathological manifestations prior to A*β* deposition. Exogenous administration of A*β* is vasoconstrictive and vessels treated with A*β* show significant endothelial cell damage with changes in the cell membrane, cytoplasm, nucleus, and other organelles [16]. Takechi et al. [28] suggested that that chronic ingestion of SFA may increase TRL-A*β* secretion and that repeated postprandial excursions may eventually disrupt BBB function. Consistent with this possibility, SFAs were shown to stimulate enterocytic A*β* abundance and released into the circulation associated with postprandial TRL and a similar phenomenon may also occur in liver [20, 35]. Moreover, diets enriched in SFA reduce high affinity clearance pathways of TRL-remnant lipoproteins and this may contribute to increased postprandial lipaemia and plasma A*β* [36, 37]. 

In this study, parenchymal apo B abundance did not correlate with plasma triglyceride concentration. Mice fed the DHA-enriched diet had comparable triglycerides to the SFA group and plasma triglycerides were greatest in SFA mice randomized to LF. Saturated fats often increase plasma NEFA concentration compared to low-fat diets, whereas DHA generally accelerates TRL clearance by facilitating LPL-mediated lipolysis [38, 39]. However, in this study, there was no significant increase in net concentration of plasma NEFA in mice randomized to the DHA diet versus those maintained on SFA alone (0.53 ± 0.05 versus 0.42 ± 0.03 mEq/L, resp.). Nonetheless, a role of fatty acids in modulating cerebrovascular integrity cannot be ruled out because fatty acid phenotype may be critically important. Many studies suggest significant vascular effects of fatty acids. Human aortic endothelial cells treated with TRL and lipoprotein lipase (LPL) were highly permeable, whilst cells treated with TRL or LPL alone were not [40]. Furthermore, LPL-mediated TRL lipolysis initiated degradation of the tight junction protein ZO-1 and induced an endothelial apoptotic cascade. 

The most significant lipid effect of randomization to DHA following chronic ingestion of SFA in this study was a twofold increase in plasma cholesterol compared to the mice maintained on the SFA diet, or mice randomied to the LF diet. A number of animal and clinical studies have shown that DHA-enriched diets can increase plasma cholesterol associated with low- and high-density lipoproteins [41–43]. Hypercholesterolemia has been associated with many vasculature abnormalities including endothelial dysfunction, decreased vascular reactivity, and enhanced expression of adhesion molecules [44, 45]. Cell culture studies suggest several mechanisms by which cholesterol may be pro-inflammatory and some of these appear to be analogous to the effects of dietary SFA. Yao and colleagues reported that excess cholesterol causes endoplasmic reticulum and mitochondrial stress that can lead to apoptosis [46]. Mitochondrial activity or microsomal processing also results in the production of oxidized lipids that trigger and exacerbate inflammatory pathways [47].

The anti-inflammatory properties of particularly the n3 and n6 fatty acids have been unequivocally demonstrated in many studies. However, the propensity for PUFA to oxidize may under some circumstances amplify oxidative stress sequelae. Diets enriched in SFA enhance oxidation as a consequence of stimulated mitochondrial function in activated macrophages [46]. Dietary SFA diminish the proper function of the cerebrovascular endothelial cells and are thereafter likely to activate astro-glial cells which encompass cerebral capillary vessels. It's plausible that enhanced interaction of plasma PUFA's in DHA-fed mice with activated inflammatory cells may be a primary mechanism by which the effects of SFA are amplified. Consistent with this possibility, Kuo et al. [48] showed a dose effect of dietary DHA on BBB permeability in mice supplemented with 12% fish oil for 6 months, versus mice fed 3% fish oil. Similarly, rats consuming fish oil exhibited increased lipid peroxidation [49, 50] and oxidative stress-induced damage of DNA in the absence of dietary antioxidants [51]. In vitro, DHA and EPA enhanced lipid peroxidation and triggered cellular apoptosis [52, 53]. 

## 5. Summary

Chronic ingestion of diets enriched in SFA commonly causes vascular dysfunction, including in capillary vessels of the brain. The effects of SFA could be described as a response-to-injury phenomenon induced by exaggerated exposure to plasma triglyceride, cholesterol, NEFA, or harmful inflammatory products of lipid metabolism, such as lipid peroxides. Many studies support a role of n3 and n6 fatty acids in the prevention of vascular, based disorders primarily via suppression of inflammatory cascades. Less clear however are the benefits of polyunsaturated oils in the presence of profound inflammation, because of the propensity to generate lipid peroxidation products. 

In an established model of cerebrovascular dysfunction induced by chronic ingestion of an SFA-enriched diet, provision of DHA amplified the harmful effects. Probable mechanisms include hypercholesterolemia and perhaps fatty acid-induced cytotoxicity. The data suggests that introduction of n3/n6 fatty acids in metabolic conditions that are characterized by heightened systemic inflammation needs to be carefully considered in the context of paradoxical detrimental effects that could occur. 

## Figures and Tables

**Table 1:** Dietary composition.

	SFA diet	LF diet	DHA diet
Calculated nutritional parameters (%)			
Protein	13.6	13.6	13.6
Total fat	20.3	4	20.3
Crude fibre	4.7	4.7	4.7
Acid detergent Fibre	4.7	4.7	4.7
Digestible energy	18.8 MJ/kg	15.1 MJ/kg	18.8 MJ/kg
% Digestible energy from lipids	40	n/a	40
% Digestible energy from protein	15	n/a	15

Calculated fat composition (%)			
Myristic acid 14:0	0.05	Trace	0.54
Pentadecanoic acid 15:0	0.01	n/a	0.16
Palmitic acid 16:0	5.16	0.2	3.26
Megaric acid 17:0	0.05	n/a	0.18
Stearic acid 18:0	7.31	0.1	0.92
Arachidic acid 20:0	0.24	n/a	0.06
Behenic acid 22:0	0.04	n/a	0.05
Tetracosanoic acid 24:0	0.03	n/a	0.05
Palmitoleic acid 16:1	0.05	Trace	0.66
Heptadecenoic acid 17:1	0.01	n/a	0.10
Oleic acid 18:1 n9	6.62	2.4	2.25
Gadoleic acid 20:1	0.01	n/a	0.18
Lenoleic acid 18:2 n6	0.67	0.8	0.23
a Linolenic acid 18:3 n3	0.05	0.4	0.09
g Linolenic acid 18:3 n6	Not detected	n/a	0.08
Arachadonic acid 20:4 n6	Not detected	Trace	0.46
EPA 20:5 n3	Not detected	Trace	2.00
DHA 22:6 n3	Not detected	Trace	8.22

The total fatty acid composition of SFA, LF, and DHA diets. Vitamin and mineral contents were balanced in all diets.

**Table 2:** Effects of various feeding regimens on plasma lipids in wild-type (C57BL/6J) mice.

	SFA 5 m	+LF 2 m	+DHA 2 m
TG (mM)	0.39 ± 0.04	0.54 ± 0.03*	0.43 ± 0.04
TC (mM)	1.45 ± 0.23	1.69 ± 0.17	3.11 ± 0.12**
NEFA (mEq/L)	0.42 ± 0.03	0.45 ± 0.03	0.53 ± 0.05
Body weight			
Final	25.65 ± 0.64	23.72 ± 0.96	20.21 ± 0.50**
Weight gain	8.41 ± 0.51	5.7 ± 1.12	3.3 ± 0.51**

Plasma total cholesterol (TC), triglycerides (TGs), and non-esterified fatty acids (NEFAs) were measured at the end of the feeding regimen in mice fed saturated fats (SFA 5 m) and SFA-fed mice switched to an LF (+LF 2 m) and DHA diet (+DHA 2 m). Final body weight and weight gain were also calculated. Data represented as mean ± standard error of mean. Means were compared with one-way ANOVA, where *P* < 0.05 considered statistically significant (*).
